# Papillary Thyroid Cancer, Macrofollicular Variant: The Follow-Up and Analysis of Prognosis of 5 Patients

**DOI:** 10.1155/2014/818134

**Published:** 2014-09-10

**Authors:** Varlık Erol, Özer Makay, Yeşim Ertan, Gökhan İçöz, Mahir Akyıldız, Mustafa Yılmaz

**Affiliations:** ^1^Division of Endocrine Surgery, Department of General Surgery, School of Medicine, Ege University, Bornova, 35100 Izmir, Turkey; ^2^Department of Pathology, School of Medicine, Ege University, Bornova, 35100 Izmir, Turkey

## Abstract

*Objective.* The main aim of this study was to comparatively analyze the recurrence and prognosis of this rare variant with the literature by analyzing the follow-up data of 5 patients diagnosed with papillary cancer macrofollicular variant. *Methods.* The demographic data, radiological and pathological data, and prognostic data of 5 patients who underwent surgery for thyroid cancer and were diagnosed with papillary cancer macrofollicular variant pathologically were retrospectively analyzed. *Results.* The mean age of patients whose mean follow-up period was determined as 7.2 years was 41, and the male/female ratio was 4/1. All patients underwent total thyroidectomy. The pathology report of 2 patients (40%) revealed macrofollicular variant of papillary microcancer, and 3 patients papillary cancer macrofollicular variant. Central dissection was performed in one patient (20%) due to macroscopic pathologic lymph node and 4 metastatic lymph nodes were reported. Also, locoregional recurrence was present in 3 out of 5 patients (60%). *Conclusions.* Although an impression of earlier and increased risk of recurrence in papillary carcinoma with macrofollicular variant has been documented, more studies with extensive follow-up times and large populations are required.

## 1. Introduction

Thyroid cancer is the most common endocrine malignancy, it accounts for less than 1% of all cancers, and the tumors usually progress slowly [1]. Papillary thyroid cancer is the most common of all thyroid cancers. It makes up 80% of all thyroid cancers [2]. Despite high survival rates, local recurrence and metastasis occur in some patients and this may require more aggressive surgical treatment. It is more common in women; the incidence varies according to different reports. Male : female ratio varies from 1 : 2 to 1 : 10. They often appear in the third and fifth decades of life [3, 4]. Papillary thyroid carcinoma exhibits a morphologically wide spectrum. Follicular variant of papillary thyroid cancer subtypes is characterized by follicles lined by cells with nuclear features of papillary carcinoma. Follicles are usually associated with small, dense colloids [5]. Macrofollicular variant of papillary carcinoma (MVPC) is a rare subtype of follicle variant, and it was first defined by Albores-Saavedra et al. in 1991 [6] and was described as encapsulated follicular variant that includes more than 50% macrofollicular in each section. Macrofollicle was defined as a follicle that is of a diameter greater than 250 *μ*m. In this study, the aim was to examine the prognostic features of MVPC by analyzing the follow-up results of 5 patients diagnosed with MVPC.

## 2. Materials and Methods

Demographic data, preoperative data, postoperative treatment methods (hormone replacement, radioactive iodine ablation therapy), locoregional recurrence rates, and prognostic factors of patients who underwent surgery due to thyroid cancer in Ege University Hospital, Department of General Surgery, Division of Endocrine Surgery, and were diagnosed with MVPC between January 2000 and January 2006 were retrospectively analyzed.

All treatment decisions were taken by an endocrine council consisting of general surgeons, endocrinologists, nuclear medicine specialists, pathologists, and radiologists, by adopting a multidisciplinary approach. In our clinic, we applied total thyroidectomy for patients diagnosed with thyroid cancer, in a way that no visible tissue would be left behind.

Locoregional recurrence was described as cases having pathological lymph nodes on ultrasonography and/or cytology and/or a new determination of a previously nonexisting tissue or presence of increased levels of thyroglobulin.

## 3. Results

The mean age of the patients included in the study was 41 (38–48). Four of them were females (80%) and 1 was male (20%). The mean follow-up duration was 7.2 years (5.3–11.2). While the reason for one patient's admission was symptoms of hyperthyroidism, no symptoms were detected in other patients. On physical examination, one of the patients had palpable thyroid nodules on right upper lobe, and another had bilateral palpable nodules. No palpable lymph nodes were detected in any of the patients. All patients had euthyroid thyroid function tests. None of the patients had thyroid cancer history in their families. None of the patients were detected to have pathological lymph nodes after ultrasonographic evaluation. All patients underwent a total thyroidectomy.

**Figure 1:**
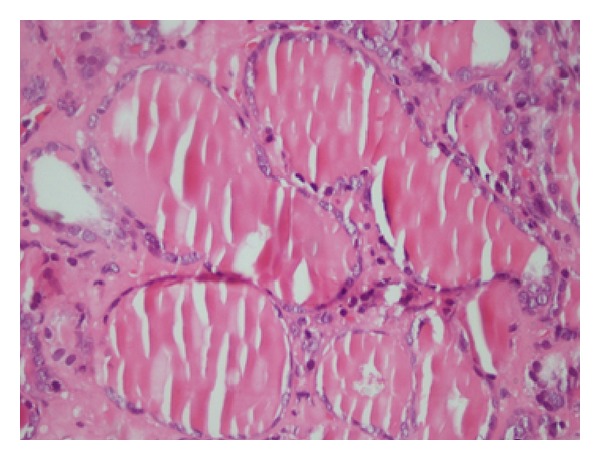
MVPC histological image (hematoxylin and eosin, ×20).

**Figure 2:**
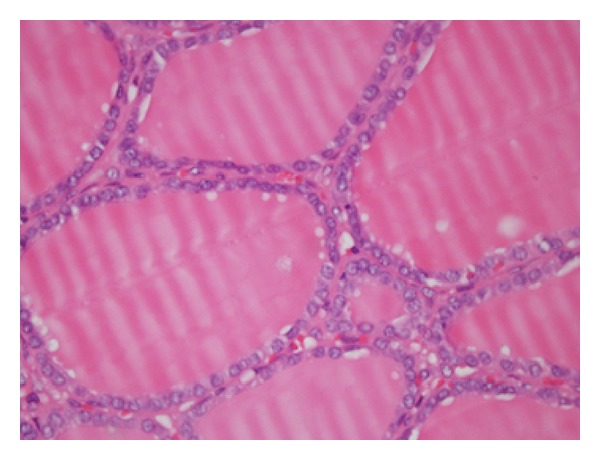
Follicular structures lined by tumor cells that include papillary carcinoma nuclear features.

In the histological examination of tumors that were observed in thyroidectomy materials, macrofollicular structures were determined in most areas and microfollicular in some others. These cells lining the follicles were of clear nuclei and showed obvious deformity of the nuclear membrane, notch structure, and sparse pseudoinclusion. It was remarkable that a portion of the cells lining macrofollicles had hyperchromatic nuclei. Also, vacuolization was spotted in colloid in the periphery of the follicle structures. With these histological findings, the tumor found in all patients was reported to be MVPC (Figures 1 and 2). More than 50% of the cross-sectional area of the tumor was composed of macrofollicles (follicles 250 *μ*m in diameter) in all patients. Pathological tumor sizes were reported as <1 cm in 2 patients, 1.1–2 cm in another two, and 2.1–3 cm in the last one. Multifocal tumors were reported in one patient. There was no tumor invasion (soft tissue, capsules, and vessels) in any of the patients. Upon detecting lymph nodes in intraoperative macroscopic pathology, one patient (20%) was applied ipsilateral central (6th level) lymph node dissection and 4 metastatic lymph nodes were reported histologically out of 11 dissected lymph nodes.

While four of the patients (80%) were applied radioactive iodine ablation (RIA) in the postoperative period, 1 (20%) was not. The patient, who was detected to have lymph node metastases, received RIA at a dose of 225 mCi, and the other three received <150 mCi. Locoregional recurrence was detected in three patients. One patient was operated due to recurrence, and others were controlled by RIA. Mortality was observed in none of the patients (Table 1).

## 4. Discussion

Papillary thyroid carcinoma is the most common malignant tumor of the thyroid gland, and it is in the group of well-differentiated tumors thanks to good prognosis. It is also the type of thyroid cancer which has the widest histological spectrum. MVPC histologically consists of follicles and cells of papillary cancer nuclear characteristics which line these follicles. While macrofollicles can also be encountered in classical papillary cancer and follicular variant, more than 50% of the tumor in macrofollicular variant consists of macrofollicles. Defining this variant is important as it can easily be mistaken with benign thyroid lesions, such as nodular goiter and follicular adenoma. Macrofollicles in MVPC are lined by tumor cells with nuclei that are large, overlapping, clear, and notched and that sometimes have cores containing pseudoinclusion [7]. Minor nuclear irregularities can also be seen in benign lesions, but this histological finding is not enough for a cancer diagnosis alone [7]. In addition, unlike benign thyroid lesions, there is a remarkable nuclear deformity in the shapes of cells. Histological diagnosis is made according to nuclear properties of tumor cells paving the macrofollicles because macrofollicles are lined by squamous cells in some MVPCs [8].

Definitions as case presentations exist in the literature, the largest series of which is the one made by Albores-Saavedra et al. in 1991 with 29 patients to define MVPC and republished the results in 1997 with the adding up new cases [9]. There are no large series of patients, but it can be said that it is more common in females than males [6]. Similarly, male/female ratio was 4/1. MVPC was reported to have a very good prognosis with its lower rate of lymph node metastasis compared to classical papillary cancer [9, 10]. Clinically, they are usually included in nonaggressive tumors, and in the study of Albores-Saavedra et al. rate of lymph node metastasis was reported as 11.8%, in their second study as 20.6%, and in another study by Evans as 7.1% (1 metastasis in 14 patients) [6, 9, 11]. In this study, lymph node metastasis rate was determined as 20%. MVPC lymph node metastases rate's being lower while it is higher in general papillary cancer is associated with the fact that it is a well-limited capsulated and having low proliferative activity [6, 9].

Contrary to the abovementioned good prognostic data, 2 out of 29 patients were reported to have capsular invasion, vascular invasion, and lung metastasis in the study of Albores-Saavedra et al. [9]. Also, in the study by Cardenas et al. 2 cases with aggressive behaviour (extrathyroid extension, lymph nodes, and bone and lung metastases) were reported [12]. In this study, distant metastasis and death were not detected in a mean follow-up period of 7.2 years, and locoregional recurrence rate was observed to be high (60%). In a study we conducted in our clinic but not yet published, 5 out of 181 patients (2.7%) diagnosed with well-differentiated thyroid cancer were detected to have MVPC. In a mean follow-up period of 7.1 years, locoregional recurrence rate of 181 patients was determined to be 22.6% (41 patients), and the locoregional recurrence rate of MVPC group in this group of patients was found to be higher (60%). In this small series, we describe two cases of microcarcinoma exhibiting macrofollicular architecture: these are two aspects generally correlated with an excellent prognosis. Regarding the clinical behavior of these cases in this series, one of the recurrent patients had a microcarcinoma. This patient, as well as the rest in this series, had no invasion reported on histopathological review. The other microcarcinoma patient had no recurrence during follow-up.

Although MVPC is said to be a variant that has a perfect prognosis and less rate of lymph node metastasis than those of classical papillary cancer, in this study the rate of lymph node metastasis was found to be 20% and locoregional recurrence rate 60%. It can be argued that the number of patients included is not enough to make definite statements about prognosis, but studies including such large groups do not exist in the literature. Special care should be given especially because one can encounter other benign thyroid diseases in fine-needle aspiration biopsy and histological evaluation.

As its histological subtypes and the clinical behaviour of these subtypes are highly variable, this definition has started to prove insufficient though papillary cancer is one of the diseases with best prognosis. For this reason, we are of the opinion that prospective prognostic studies that evaluate all papillary cancer subtypes together with higher number of patients are a need today.

## Figures and Tables

**Table 1:** General characteristics of patients.

	*n* (%)
Age	
≤45	4 (80%)
>45	1 (20%)
Gender	
Female	4 (80%)
Male	1 (20%)
Ultrasonographic nodule size	
≤1 cm	0
1.1–2 cm	3 (60%)
2.1–3 cm	2 (40%)
>3 cm	0
Histopathological tumor size	
≤1 cm	2 (40%)
1.1–2 cm	2 (40%)
2.1–3 cm	1 (20%)
3.1–4 cm	0
>4 cm	0
Surgical technique	
Total thyroidectomy	5 (100%)
Histopathology	
MVPC	5 (100%)
Lymph node metastasis	1 (20%)
Tumor invasion	0
Postoperative treatment	
Radioiodine ablation	4 (80%)
≤150 mCi	3 (75%)
>150 mCi	1 (25%)
Locoregional recurrence	3 (60%)
Radioiodine ablation (>150 mCi)	1 (33.3%)
Radioiodine ablation (≤150 mCi)	2 (66.6%)
Mortality	0
